# Comparison of central corneal thickness measurements with the Galilei dual Scheimpflug analyzer and ultrasound pachymetry

**DOI:** 10.4103/0301-4738.67045

**Published:** 2010

**Authors:** Jeevan S Ladi, Nitant A Shah

**Affiliations:** Dada Laser Eye Institute, Pune, India

**Keywords:** Central corneal thickness, dual Scheimpflug analyzer, ultrasound pachymetry

## Abstract

**Purpose:**

To compare corneal pachymetry assessment by the Galilei dual Scheimpflug analyzer with that done by ultrasound (US) pachymetry.

**Materials and Methods:**

Forty six patients (92 eyes) were subjected to corneal pachymetry assessment by Galilei dual Scheimpflug analyzer and US. All the readings were taken by a single operator. Intraoperator repeatability for the Galilei was assessed by taking 10 readings in one eye each of 10 patients. To study the interoperator reproducibility for the Galilei, two observers took a single reading in both the eyes of 25 patients.

**Results:**

The mean central corneal thickness (CCT) measured by US was 541.83 ± 30.56 μm standard deviation (SD) and that measured by Galilei was 541.27 ± 30.07 μm (SD). There was no statistically significant difference between both the methods (*P* < 0.001). The coefficient of repeatability was 0.43% while the coefficient of reproducibility was 0.377% for the Galilei.

**Conclusion:**

Objective, noncontact measurement of the CCT with the Galilei dual Scheimpflug analyzer was convenient, had excellent intraoperator repeatability and interoperator reproducibility, and findings were similar to those obtained with standard US pachymetry.

Accurate measurement of central corneal thickness (CCT) has assumed significance in ophthalmic practice. It is important in corneal refractive surgery, especially laser assisted *in situ* keratomileusis (LASIK) which is the most commonly performed corneal procedure currently.[1] The CCT allows determination of the amount of stromal ablation which can be safely carried out minimizing the risk of iatrogenic keratectasia.[1] CCT is also used in glaucoma practice to modify the intraocular pressure reading for accuracy.[2]

Applanation ultrasound (US) pachymetry is currently the most often method used for the measurement of CCT. This method has been reported to have a high degree of intraoperator and interoperator reproducibility.[3] However, placement of the probe on the corneal center is subjective and operator-dependent errors due to off-center placement (leading to thicker measurements) are a possibility.[4,5] Errors can be caused by indentation leading to slightly thinner readings.[6] In addition, disadvantages like patient discomfort, epithelial damage and risk of infection exist. Hence, it is not surprising that noncontact techniques to measure CCT are gaining popularity. Partial coherence interferometry (PCI),[7] optical coherence tomography (OCT),[8] scanning slit tomography/pachymetry[9] are some of the optical techniques introduced to measure CCT.

The Galilei dual Scheimpflug analyzer (Ziemer, Switzerland), uses two rotating Scheimpflug cameras combined with a Placido disk to image the anterior segment of the eye. It is a noncontact instrument which provides data on anterior and posterior corneal topography, complete corneal pachymetry, lens densitometry and two-dimensional and three-dimensional anterior segment imaging. The purpose of this study was to report our initial experience in measuring CCT with this new device and compare it with the gold standard US pachymetry.

## Materials and Methods

Forty six patients (92 eyes) who presented to our center were prospectively studied. Patients with ocular disease (other than refractive error), contact lens wearers and those with a history of previous eye surgery were excluded. All patients were subjected to a comprehensive ophthalmic examination including vision, refraction, slit lamp examination, CCT measurement with two methods, followed by measurement of intraocular pressure and dilated fundus examination.

CCT measurements were first taken on the Galilei analyzer. Three readings were taken for each eye. A gap of 1 minute was given after each reading and the alignment was freshly done each time. Following this, the cornea was anesthetized with topical 0.5% proparacaine and five readings were taken with US pachymetry (Echorule, Biomedix, Bangalore, India). All the readings on the Galilei and US pachymeter were taken by a single trained optometrist.

Galilei measurements were obtained as per the manufacturers’ instructions. The patient was comfortably seated with chin fully placed on the chin rest and forehead against the strap. The patient looked at the target (red spot) and was allowed to blink. The device was brought in focus by aligning the measurement head of the Galilei. Alignment was considered correct when the red cross (cross hair) passed through the white spots and the single red line touched the corneal epithelium. The iris was seen in sharp focus on the screen. The patient was asked to blink once, open the eye wide and the reading was taken. The time taken for scanning was 2–3 seconds.

US pachymetry readings were taken by aligning the probe as perpendicularly as possible on the central cornea. Five readings were taken. The highest and lowest values were excluded and the mean of the remaining three were used for the analysis.

The repeatability of the Galilei analyzer was studied. Ten successive scans were obtained by a single operator in one eye each of 10 patients. An interval of 1 minute was given between two readings and alignment was freshly done each time.

Interoperator reproducibility was studied in the following manner. Two operators took a single reading of the right and left eyes of 25 patients. An interval of 5 minutes was given between the two operators.

All the data were analyzed using the SPSS computer program for Windows (Version 11.5, SPSS, Inc., Chicago, Illinois).

### Comparison of CCT

The results were entered as mean ± standard deviation (SD). All the data were tested for normality using skewness (acceptable range of normality is between –1 and +1) and kurtosis (acceptable range of normality is between –1 and +1). The statistical agreement between the two methods was assessed using interclass correlation coefficient. Bland–Altman plot was used to test the agreement between the two measuring techniques. A *P* value of less than 0.05 was considered as statistically significant.

### Study of repeatability and reproducibility

The data were entered as mean (SD). All the data were tested for normality before analysis. In order to assess the pairwise statistical difference between the averages of two observers, the paired *t*-test was adopted. For each patient, the coefficient of repeatability was defined as the SD of the difference from the mean of the repeat measurements, divided by the mean response. The coefficient of interobserver reproducibility was defined as the SD of difference between the pairs of the measurements obtained by two observers, divided by the average of the means of each pair of observation. We have used definitions for repeatability and reproducibility adopted by the British Standards Institution as recommended by Bland and Altman.[10] A *P* value less than 0.05 was considered as statistically significant.

### Results

The results of repeatability study are shown in Table 1. The mean coefficient of repeatability for 10 readings in 10 patients was 0.43% which demonstrates good reliability.

**Table 1:** Coefficients of repeatability for 10 patients using the Galilei analyzer

Patient ID	Coefficient of repeatability (%)
1	0.15
2	0.19
3	0.35
4	0.25
5	0.32
6	0.44
7	0.79
8	0.21
9	1.29
10	0.34
Mean	0.43

**Figure 1:**
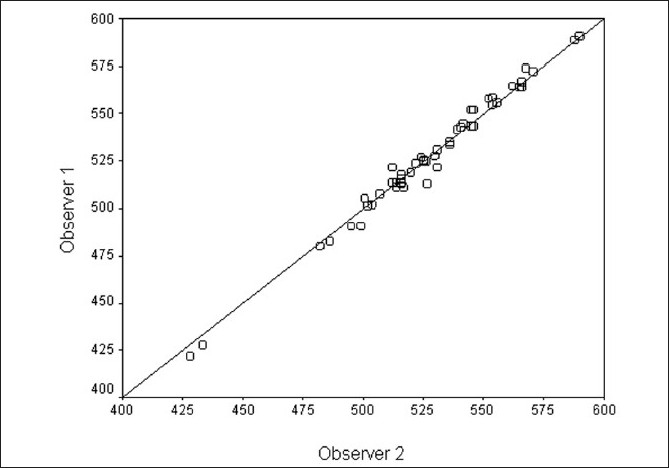
Scatter plot for comparing CCT between two observers with no change line. *X* and *Y* axes show CCT values measured by observer 2 and observer 1, respectively, in micrometers (either eye, *n* = 50)

The mean CCT value taken in 50 eyes using the Galilei for observer 1 was 527.8 ± 33.7 μm, while it was 527.9 ± 31.9 μm for observer 2. There was no statistically significant difference between them. (*P* = 0.739). The coefficient of interoperator reproducibility for the Galilei was 0.377%. This and the scatter plot depicted in Fig. 1 indicate good interobserver reproducibility.

The mean CCT obtained by US pachymetry for 92 eyes was 541.83 ± 30.56 μm (skewness –0.417 and kurtosis –0.312). The mean CCT by Galilei for 92 eyes was 541.27 ±30.07 μm (skewness –0.3 and kurtosis –0.151). Mean difference between the two methods was 0.55 μm. The interclass correlation coefficient was 0.978. There was no statistically significant difference between the CCT readings taken by Galilei and US (*P* < 0.001). Correlation between fellow eyes has been adjusted in the statistical analysis using multivariate linear regression.

**Figure 2:**
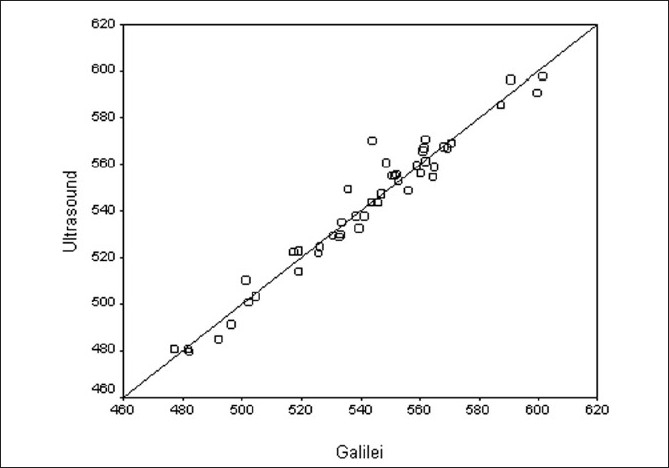
Scatter plot for comparing CCT between two methods with no change line. *X* axis: measurement of CCT with Galilei in micrometers. *Y* axis: measurement of CCT with US in micrometers

**Figure 3:**
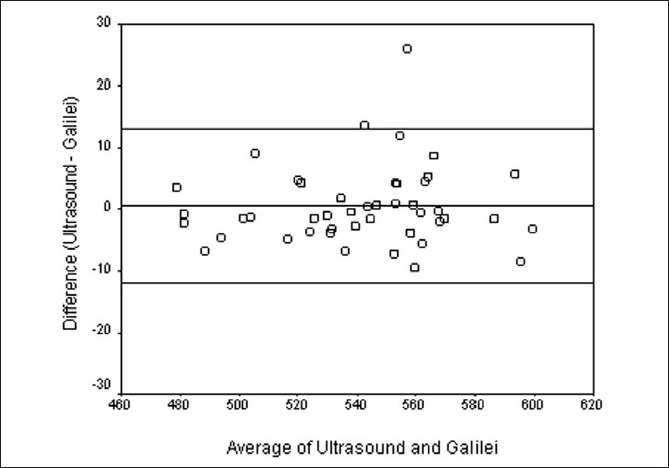
Bland–Altman Plot comparing the two modalities of measurement, viz., comparison between Galilei and ultrasound pachymetry

Fig. 2 is a scatter plot with a no change line to compare the CCT measurement by the two methods. Fig. 3 is a Bland–Altman plot and shows good agreement between Galilei and US. The 95% limits of agreement were –11.93 to +13.03. The plot indicates that there is no systematic bias between the two methods (*P* = 0.612 by linear regression method between difference and average by two methods.)

## Discussion

Noncontact techniques like PCI and OCT have been shown to have high intraoperator repeatability and interoperator reproducibility in literature.[7,8] This is important for a new instrument to gain widespread acceptance.

Anterior segment imaging using Scheimpflug analyzers was reported initially using the EAS-1000 (Nidek Co. Ltd.).[11] This model acquired a single image of the anterior segment. The Pentacam was an improvement over this, having a single rotating camera which acquires upto 50 images in a scan. The Galilei analyzer has two rotating Scheimpflug cameras, 180° apart, which record simultaneously. This helps to compensate for errors associated with scans at an oblique angle and increases the accuracy for measuring not only the central cornea but also the peripheral cornea (in spite of eye micromovements which are unavoidable).

Previous studies have shown the coefficient of repeatability to be 0.84% for the Pentacam, 0.33% for the optical low-coherence reflectometer (OLCR) pachymeter, and 0.71% for standard US pachymetry.[12] The OCT has been shown to have a coefficient of 2%.[8] In our study, the coefficient of repeatability for the Galilei was 0.43%. Menassa *et al*.[13] have reported a remarkably low intraobserver and interobserver variation, this being the only report on the Galilei in the literature so far.

The coefficient of interobserver reproducibility was 0.37% in our study. This coefficient has been reported to be 1.10% for the Pentacam and 0.59% for the OLCR,[12] while it was 0.18% for OCT.[8] Another study by Lackner *et al*.[14] has shown the reproducibility by Pentacam to be the highest between Pentacam, Orbscan and US. Menassa *et al*.[13] have reported better interobserver reproducibility with the Galilei compared to Orbscan II.

Previous studies have reported that CCT measurements in healthy eyes by different instruments can be significantly different. In a study of 34 normal eyes, Modis *et al*.[9] have reported mean CCT to be 547 ± 49 μm by noncontact specular microscopy, 580 ± 43 μm by US, 602 ± 59 μm by Orbscan and 640 ± 43 μm by contact specular microscopy. Chakrabarti*et al*.[15] have reported Orbscan measurements to be 28 μm higher than those of US. Due to the considerable difference, Orbscan manufacturers recommended incorporation of an acoustic factor of 0.92. This was calculated to compensate for the effect of tear film whose thickness gets included while taking CCT measurements. Indeed after applying the correction factor, Wong *et al*.[16] have reported CCT to be 555.96 ± 32 μm with Orbscan and 555.11 ± 35 μm with US. Also, Suzuki*et al*.[17] have reported CCT to be 548.1 ± 33 μm with US and 546.9 ± 35.4 μm with Orbscan. Ho *et al*.[18] have recommended the use of a custom acoustic factor specific to the study site. They have used an acoustic factor of 0.89 for Orbscan II to obtain readings compatible with US. Similarly, Menassa *et al*.[13] have used a correction factor of 0.96 for Orbscan II. Lackner *et al*.[14] have reported an underestimation of 9.8 μm with the Pentacam as compared to US. Barkana *et al*.[12] have reported an underestimation of 6.09 μm with the Pentacam as compared to US, which was statistically significant.

Menassa *et al*.[13] have reported a mean difference of 6.8 μm between CCT readings by Galilei (551.7 ± 36.6) and US (558.5 ± 38.4), which was statistically significant. In our study, comparison of CCT measurements between the Galilei and US has shown that they differed by a mean of only 0.55 μm. This difference is not statistically significant. The 95% limits of agreement showed that the difference in measurements between the two methods was between –11.93 and +13.03 μm. This is remarkably close to the range of ±11 μm for the diurnal pachymetric variation of CCT. Noncontact methods would include the tear film thickness (5–7 μm) in CCT measurement. However, as suggested by Ho *et al*.,[18] US may also overestimate CCT due to the effect of analgesic eyedrops used before pachymetry. These can cause epithelial edema leading to increased corneal thickness. Further studies would be required to find out which method ultimately gives us the true CCT.

In conclusion, the Galilei analyzer can measure CCT with an excellent repeatability and reproducibility. This noncontact method of examination can thus be delegated to nonmedical personnel like technicians or optometrists. The pachymetry readings with Galilei showed good correlation with those of US pachymetry and there was no statistically significant difference between them.

To the best of our knowledge, this is the first study using the Galilei dual Scheimpflug analyzer in Indian Asian eyes.
